# Do informed consent documents for chiropractic clinical research studies meet readability level recommendations and contain required elements: a descriptive study

**DOI:** 10.1186/s12998-014-0040-9

**Published:** 2014-12-10

**Authors:** Elissa Twist, Dana J Lawrence, Stacie A Salsbury, Cheryl Hawk

**Affiliations:** Palmer Center for Chiropractic Research, 741 Brady Street, Davenport, IA 52803 USA; Palmer College of Chiropractic, 1000 Brady St., Davenport, IA 52803 USA; Logan University, 1851 Schoettler Rd., Chesterfield, MO 63017 USA

**Keywords:** Bioethics, Chiropractic, Informed consent, Clinical trials, Manual therapies, Ethical review

## Abstract

**Background:**

Informed consent documents (ICD) in research are designed to educate research participants about the nature of the research project in which he or she may participate. United States (US) law requires the documents to contain specific elements present and be written in a way that is understandable to research participants. The purpose of this research is to determine if ICDs from randomized controlled trials conducted at chiropractic colleges meet recommended readability standards and contain the 13 content items required by US law.

**Methods:**

This study was approved by Palmer College of Chiropractic’s IRB #2012-12-3-T and was conducted between December 3, 2012 and February 14, 2013. We contacted the research directors of five chiropractic colleges that have received federal funding supporting their clinical research. A total of 13 informed consent documents from four chiropractic colleges were analyzed using the Flesch-Kincaid measurement. We assigned a grade-level readability score to the document based on the average of three separate grade level scores conducted on the three largest uninterrupted blocks of text. Content of the 13 ICDs was assessed using a 13-element checklist. A point was given for every element present in the document, giving a score range of “0, no elements are present”, to “13, all elements are present.”

**Results:**

The mean Flesch-Kincaid grade level readability was 10.8 (range 7.2 -14.0). Our sample had a mean readability score 2.8 grade levels above the generally-accepted US average reading level. Content varied among the 13 informed consent forms, ranging from only nine elements present in one document to all 13 required in five documents. Additionally, we collated the risks presented in each document.

**Conclusion:**

These results strongly suggest that chiropractic clinical researchers are not developing ICDs at a readability level congruent with the national average acceptable level. The low number of elements in some of the informed consent documents raises concern that not all research participants were fully informed when given the informed consent, and it may suggest that some documents may not be in compliance with federal requirements. Risk varies among institutions and even within institutions for the same intervention.

## Background

Informed consent in research is designed to help educate a research participant about the nature of the research project in which he or she may participate. This process empowers the participant to make an autonomous, freely chosen decision. Beauchamp and Childress note that informed consent can only be given if a research participant is competent, receives complete disclosure about the intervention, understands the disclosure, acts on his or her own will, and consents to the intervention [1]. Research participants are only able to exercise their decision-making autonomy if the information in the informed consent is comprehensible to them. To give participants adequate disclosure about a research project, United States (US) law requires the informed consent documents (ICDs) used in human subject research to provide specific information to research participants [2]. The US National Institute of Justice has identified 13 elements that must be included in the informed consent document (Table 1) [3]. Additionally, under US law ICDs need to be in language understandable to the research participant [3]. A National Cancer Institute working group recommended that ICDs for clinical trials should be written at or below an 8th grade reading level to increase the understandability of the document for more participants [4]. Paasche-Orlow, Taylor, and Brancati examined 114 web sites of US medical schools for institutional review boards’ (IRB, is a school’s research ethics committee), readability standards and informed consent form templates [5]. Average readability level for these informed consent form templates were written at a 10.6 grade reading level. The authors concluded that, for these medical schools, the language in the IRB informed consent templates often did not meet readability standards set forth by their schools’ IRBs [5].

**Table 1:** **Elements of informed consent** [3]

1.	A statement that the study involves research
2.	The names of the funding agencies
3.	An explanation of the purposes of the research
4.	The expected duration of the subject's participation
5.	A description of the procedures to be followed
6.	Identification of any procedures which are experimental
7.	A description of any reasonably foreseeable risks or discomforts to the subject
8.	A description of any benefits to the subject or to others which may reasonably be expected from the research
9.	A disclosure of appropriate alternative procedures or courses of treatment, if any, that might be advantageous to the subject
10.	A statement describing the extent of the confidentiality of records
11.	For research involving more than minimal risk, an explanation as to whether any compensation, and an explanation as to whether any medical treatments are available, if injury occurs and, if so, what they consist of, or where further information may be obtained
12.	An explanation of whom to contact for answers to pertinent questions about the research and research subjects' rights, and whom to contact in the event of a research-related injury to the subject
13.	A statement that participation is voluntary, refusal to participate will involve no penalty or loss of benefits to which the subject is otherwise entitled and the subject may discontinue participation at any time without penalty or loss of benefits, to which the subject is otherwise entitled.

Researchers have explored the role of informed consent in chiropractic practice [6], as well as the content of ICDs in chiropractic college outpatient clinics [7]. Other investigators have examined chiropractic college faculty understanding of their institutions’ informed consent policies [8], research assistants’ experiences delivering informed consent to participants in chiropractic research studies [9], and the reporting of informed consent and ethics approval in chiropractic research journals [10]. No information has been published about informed consent readability and content in the ICDs used for clinical research studies conducted in chiropractic colleges. The information gained in this study will help to identify if areas of readability and content improvement are needed in the ICDs of chiropractic clinical studies, as well as to identify what those changes more specifically may require.

The purpose of this study was to determine if the informed consent documents used in randomized controlled trials from five chiropractic colleges (of which four participated) meet average 8^th^-grade readability standards and contain the 13 elements of informed consent required by US law. The specific aims of the study were to: (a) determine grade-level readability level of chiropractic clinical trial ICDs obtained from four chiropractic colleges that have established clinical research programs, by using the Flesch-Kincaid readability scale, and (b) perform a content analysis of chiropractic ICDs obtained from four chiropractic colleges that have established clinical research programs.

## Methods

We conducted a descriptive study on a convenience sample of 13 ICDs between December 3, 2012 and February 14, 2013. The Palmer College of Chiropractic IRB ruled this project exempt from full IRB review, assurance #2012-12-3-T. The principal investigator (ET) sent an invitational letter to the research director of five US chiropractic colleges known to have received federal funding to conduct clinical research to explain the purpose, aims, methods, confidentiality procedures, and voluntary nature of the study. The letter explained that this descriptive study would analyze the ICDs for their readability levels and required content. We asked interested research directors to send digital copies of ICDs from clinical research studies conducted with human research participants at his or her institution. No other limits were set for the inclusion of the ICDs. A reminder letter was sent two weeks after the first to any institution that had not yet responded.

The readability score of each ICD was measured following the methods described by Paasch-Orlow [5]. The documents were analyzed using the Flesch-Kincaid (F-K) readability scale. The Flesch-Kincaid grade level calculator uses the formula (.39 X ASL) + (11.8 X ASW). In this formula, ASL is the average sentence length, defined as the number of words divided by the number of sentences. ASW is the average number of syllables per word, defined as the number of syllables divided by the number of words [11]. The analysis was conducted in Microsoft Word 2010 (Microsoft Corporation, Redmond, WA, US). Word count and F-K grade level were determined for every uninterrupted block of text in the ICD. The Flesch-Kincaid grade level was given to the document based on the average of three separate measurements conducted on the three largest uninterrupted blocks of text. This then gave an overall document score. We also conducted a sub-section Flesch-Kincaid analysis of the following sections, if present, to identify the sections of the ICD that were at a high readability level: 1) purpose, 2) eligibility, 3) intervention, 4) risks, 5) protections, 6) benefits, 7) voluntary, and 8) study alternatives.

The ICDs were also analyzed to examine whether they contained the 13 items that the National Institute of Justice website lists as required elements (Table 1) [3]. A checklist was created listing each element required by law. If the document contained the element, that box was checked. The check marks were tallied and each ICD was given a score ranging from 0–13. A score of 13 would indicate that all required elements were present, while a score of 0 indicated that no required items were present in the document. The principal investigator (ET) and another member of the research team (SS) independently assessed each document for the presence of the 13 required elements. Elements determined not to be present in the ICDs were then listed and categorized. The investigators met to discuss their scoring and harmonize their results when disagreements were found in scoring.

We additionally looked at how the risks printed in the ICDs were described. Risks of spinal manipulation presented in the informed consent document were extracted and listed for the type of treatment used in that study, such as cervical or lumbar manipulation. Once the risks were listed according to the area of spinal manipulation preformed in the research, we tallied up the number of studies that presented each risk. Data were entered into SPSS 20.0 (SPSS Inc., Chicago, IL, US). The data were summarized using descriptive statistics, including counts, central tendency, and dispersion.

## Results

### Response rate

Research directors from four of the five chiropractic colleges contacted submitted a total of 13 informed consent documents, with one college submitting one ICD, and the others submitting three, four and five ICDs. Eight ICDs were from studies of lumbar manipulation, three involved cervical manipulation, and two were for other procedures. The sample included ICDs for studies conducted by eight different principal investigators, with five of the 13 ICDs from federally funded projects. The ICDs ranged from four to 16 pages in length, with a mean of 6.9 (SD 3.5) pages and word counts ranging from 1228 to 5314, with a mean of 2765 (SD 1113.3).

### Readability levels

The overall mean Flesch-Kincaid grade level for the 13 ICD was 10.8, with a standard deviation of 2.2 (range 7.2-14.0). The Flesch-Kincaid readability analysis of ICD sub-sections, also all measured above the recommended 8th-grade reading level (Table 2). Of note, statements on the study purpose (mean 11.8, SD 4.5), risks (mean 11.5, SD 2.5), and benefits (mean 11.1, SD 4.6) were written at higher grade levels than the overall ICD, with the statement of study protections (presented in only four ICDs) as the only section written close to the recommended 8th grade level (mean 8.4, SD 0.4).

**Table 2:** **Flesch-Kincaid grade reading level of chiropractic research informed consent documents**

**Informed Consent Section**	**Flesch-Kincaid Grade Reading Level Mean(SD)**
Document* (n = 13)	10.8 (2.2)
Purpose section (n = 13)	11.8 (4.5)
Eligibility section (n = 6)	9.3 (4.1)
Intervention section (n = 13)	9.6 (2.5)
Risk section (n = 13)	11.5 (2.5)
Protections section (n = 4)	8.4 (0.4)
Benefits section (n = 13)	11.1 (4.6)
Voluntariness section (n = 13)	10.0 (2.7)
Study alternatives section (n = 10)	10.2 (2.1)

*The average of the 3 largest uninterrupted sections of the informed consent document.

SD- Standard Deviation.

### Required consent elements

On initial review, one investigator found the number of elements present in the ICDs ranged from nine to all 13. The other investigator determined the elements present in the documents varied from seven elements to all 13. The two reviewers met to discuss and harmonize their results. Table 3 summarizes these results. The two ICDs containing only ten elements were both missing an explanation about which procedures were experimental, as well as a disclosure of appropriate alternative procedures or treatments. One of the documents did not identify the funding agencies, and the other did not provide an explanation of compensation or medical treatment if injury occurs. All of the three documents containing 11 elements failed to disclose funding agencies. Two of these documents did not identify the procedures that were experimental, and one of them did not identify appropriate alternative procedures. All four of the ICDs containing 12 elements did not identify the procedures that were experimental.

**Table 3:** **Number of elements present in the informed consent documents (n = 13)**

**Number of ICDs**	**Number of Elements**
2	10
3	11
4	12
4	13

### Risks identified

Overall, 11 of the 13 ICDs came from spinal manipulation trials. Table 4 lists the risks of spinal manipulation that we extracted from 11 ICDs, by area of manipulation. Risks and discomforts described in the ICDs ranged from “no risks involved in study participation” to more severe risks such as cauda equina syndrome, stroke and fracture. Nine of the documents described muscle and joint soreness or stiffness as a risk associated with chiropractic care. A smaller number described more serious risks such as cauda equine syndrome (three of the eight lumbar studies). Stroke was mentioned as a risk factor for two of the three cervical spine manipulation studies.

**Table 4:** **Statements of risks in the informed consent documents (ICDs) for spinal manipulation (n-11)**

**Area of spinal manipulation**	**Risk described**	**Number of ICDs including risk**
Cervical (n = 3)	Mild discomfort	1
	Falling from treatment table	1
	Light headedness	2
	Dizziness	2
	Flushed feeling	1
	Neck/upper back soreness or stiffness	1
	Headache	1
	Muscle tightness	1
	Fatigue	1
	Increased neck pain symptoms	1
	Neck pain	1
	Muscle and ligament sprain/strain	1
	Injuries to the spinal discs	1
	Neurologic impairment	1
	Bone fracture	1
	Spinal fractures	1
	Stroke	2
Lumbar (n = 8)	No risks or discomforts	1
	Falls from the table	1
	Fatigue	1
	Light-headedness	1
	Sweating/flushed feeling	1
	Dizziness	1
	Increase in current symptoms	3
	Muscle/joint soreness	5
	Transient discomfort/soreness in the back or lower extremities	3
	Worsening of symptoms in the case of disc herniation	1
	Injury to shoulder	1
	Muscle and ligament sprain/strain	1
	Disc injury	1
	Bone fracture	4
	Stroke	1
	Cauda equina syndrome	3

## Discussion

Informed consent documents in chiropractic research have not been studied despite the important role they play in participant education. The results of this study give some insight into the readability level of chiropractic informed consent documents. This study also highlights whether or not all required elements are present in those documents.

In 1998, the National Work Group on Literacy and Health estimated that 40 to 44 million Americans had low-level reading skills [12]. The results of that study highlight the importance of lowering readability levels of written documents in order to communicate with a large portion of Americans. We chose to use the 8^th^-grade readability level as a reference point because of its general acceptance as an appropriate readability level based on the National Cancer Institute’s recommendations [5]. Informed consent documents were not compared to their institution’s recommended readability level even though this varies from institution to institution and by study.

The mean grade reading level for the 13 ICDs was 10.8, or nearly three grade reading levels above the referent 8th grade level. This may be a problem for chiropractic researchers. This mean readability score could be an indication that research participants were not given the information they need in a way that is understood by them. The understandability of a document is more complex than readability level of that document alone, but it is one way to increase ease of understanding for research participants [5].

Bullet points, font size, bold face, spelling errors and white space- that is, design elements- in the informed consent document can also influence readability of a document [13]. The ‘look’ of each ICD in this study varied greatly. Some documents were presented in an inviting and visually pleasing way. Yet other ICDs were visually confusing with small print and little white space, or very inconsistent formatting throughout the document. There is a growing body of information related to ease of readability [14]; we recommend that principal investigators adapt this knowledge to the design and lay-out of future ICDs.

Our content analysis of the 13 required elements highlighted problems with the information chiropractic researchers are providing research participants. Over half of the ICDs did not contain all 13 required elements. Some informed consent documents had required element statements or headings but were so vague or poorly described that it was difficult for investigators doing the analysis to decide if the element was present in the document. Having all of the information that is federally required available and having it expressed in a complete way in the informed consent document needs to be closely addressed by all chiropractic researchers. In contrast, some ICDs went into extreme detail describing some of the elements. A previous focus group study noted that obtaining consent when too much information is presented may also be problematic for research participants and research staff [9].

Generally, information was described more clearly and completely when it was apparent, by viewing multiple forms from the same institution, that the institution had a template for the informed consent document. A National Cancer Institute task force offered a simplified guidance on ICDs for oncology researchers [5]. The chiropractic research profession may benefit from forming a committee tasked in a similar way to standardize a template and offer a standardized guidance for chiropractic researchers to assist them with creating complete and simplified documents to provide to research participants. Even if whole profession standardization is not realistic or desired, clinical researchers in the US may use the information presented in this study to assess that the forms they present to research participants meet federal guidelines.

The risks described in the ICDs in this study, largely musculoskeletal complaints, were similar to the adverse events reported in clinical studies of spinal manipulation or mobilization of the neck or low back [15,16]. However, the risks presented for spinal manipulation of the same regions varied greatly between ICDs from different colleges and even between ICDs for studies within a single college. This discrepancy poses a unique issue of what risks chiropractic researchers should present to the research participant. The US government stipulates that a list of risks that can reasonably be seen as related to the research be presented to participants [17]. Our study addresses this as a possible issue in the proper consent of research participants warranting further investigation.

### Limitations

Important limitations need to be noted with this study. First, the study results are based on a convenience sample. We invited chiropractic research directors to send us informed consent documents after giving them a general explanation of the purposes and procedures of this study. This allowed them to hand select ICDs they wanted us to see. Second, this is a small sample size from a limited number of chiropractic institutions making the results hard to generalize across chiropractic clinical studies, particularly those conducted outside the US where ethics review boards may require different readability levels or research disclosure elements.

## Conclusion

Discovering and discussing the current shortcomings in the informed consent process for chiropractic research is a first step in implementing change that could make a positive difference in educating a research participant. Our findings suggest there is room for improvement. First, efforts should be made to ensure that consent documents are written at a readability level the average American adult can understand. Second, both chiropractic research investigators and members of institutional review boards should ensure that all elements required by law are present. There are two reasons for this: compliance with the law itself, and ensuring that participants are provided the necessary information needed to make a truly informed decision. Finally, thought needs to be given to fully understanding the risks involved in chiropractic research. There was a lack of consistency related to what risks were reported to participants, especially for cervical adjusting where one study might mention the risk of stroke while a second did not.

The results of this investigation suggest that there is a need to understand how institutional review boards in chiropractic colleges review the projects submitted to them. This is the next step of our own research. We are now in process of surveying all chairs of IRBs based in chiropractic institutions in the United States. We hope to cast further light on the process of evaluation used by IRBs that review chiropractic and manual therapy research.
